# The Japanese Catheter Ablation Registry (J‐AB): Annual report in 2020

**DOI:** 10.1002/joa3.12772

**Published:** 2022-08-27

**Authors:** Kengo Kusano, Teiichi Yamane, Koichi Inoue, Misa Takegami, Yoko M. Nakao, Michikazu Nakai, Koshiro Kanaoka, Reina Tonegawa‐Kuji, Koji Miyamoto, Yu‐ki Iwasaki, Seiji Takatsuki, Kohki Nakamura, Yoshitaka Iwanaga, Wataru Shimizu, J‐AB registry investigators

**Affiliations:** ^1^ Department of Cardiovascular Medicine National Cerebral and Cardiovascular Center Osaka Japan; ^2^ Division of Cardiology, Department of Internal Medicine The Jikei University School Minato‐ku Tokyo Japan; ^3^ Cardiovascular Division National Hospital Organization Osaka National Hospital Osaka Japan; ^4^ Department of Preventive Medicine and Epidemiology National Cerebral and Cardiovascular Center Suita Japan; ^5^ Center for Cerebral and Cardiovascular Disease Information Open Innovation Center, National Cerebral and Cardiovascular Center Suita Japan; ^6^ Department of Cardiovascular Medicine Nippon Medical School Bunkyo‐ku Japan; ^7^ Department of Cardiology Keio University School of Medicine Shinjuku‐ku Japan; ^8^ Division of Cardiology Gunma Prefectural Cardiovascular Center Maebashi Japan

**Keywords:** catheter ablation, complication, J‐AB, REDCap, registry

## Abstract

The Japanese Catheter Ablation (J‐AB) registry, started in August 2017, is a voluntary, nationwide, multicenter, prospective, observational registry, performed by the Japanese Heart Rhythm Society (JHRS) in collaboration with the National Cerebral and Cardiovascular Center using a Research Electronic Data Capture system. The purpose of this registry is to collect the details of target arrhythmias, the ablation procedures, including the type of target arrhythmias, outcomes, and acute complications in real‐world settings. During the year 2020, we have collected a total of 84 591 procedures (mean age of 65.8 years and 66.6% male) from 466 participant hospitals. Detailed data were shown in Figures and Tables.

Catheter ablation has become an established therapy for the management of various cardiac arrhythmias and the procedure number has been dramatically increasing. However, little is known about the details of target arrhythmias, the ablation procedures, including the type of target arrhythmias, outcomes, and acute complications in real‐world settings.

There are several preceding registries of catheter ablation, but the majority of which collected data from selected centers and/or selected arrhythmia and/or specified months to reveal the current status of ablations.^1^, ^2^, ^3^ Accordingly, we conducted a nationwide, multicenter. Prospective, observational registry in Japan, named the Japanese Catheter Ablation (J‐AB) registry, aiming to register all catheter ablation cases in Japan.^4^ This registry has been performed by the Japanese Heart Rhythm Society (JHRS) in collaboration with the National Cerebral and Cardiovascular Center using a Research Electronic Data Capture (REDCap) system. This study has been performed under the approval of the Institutional Review Board (IRB) of the National Cerebral and Cardiovascular Center (M28‐114‐7, approved on Dec 21, 2016), Japan, along with the IRBs of all participating hospitals. All participants were provided informed consent either by a written paper or in an optout fashion and could withdraw their consent at any time. This study was also registered in the UMIN Clinical Trial Registry (UMIN 000028288) and ClinicalTrials.gov (NCT03729232). This J‐AB registry started in August 2017, and since then the number of participating hospitals has increased to over 400 at the end of 2019. Annual data during the year 2018 and 2019 has been already reported,^5^, ^6^ and now we report here the annual report of the results during the year 2020. Figure 1 showed that the cumulative number of registered hospitals and patients during the year 2020. Figure 2 showed that the number and rate of the target arrhythmias. AF procedure was the most common (74.8% of all ablation procedures) in 2020. Patient characteristics, acute outcomes, and acute complications of all and AF procedures were shown in Tables 1, 2, 3, respectively.

**FIGURE 1:**
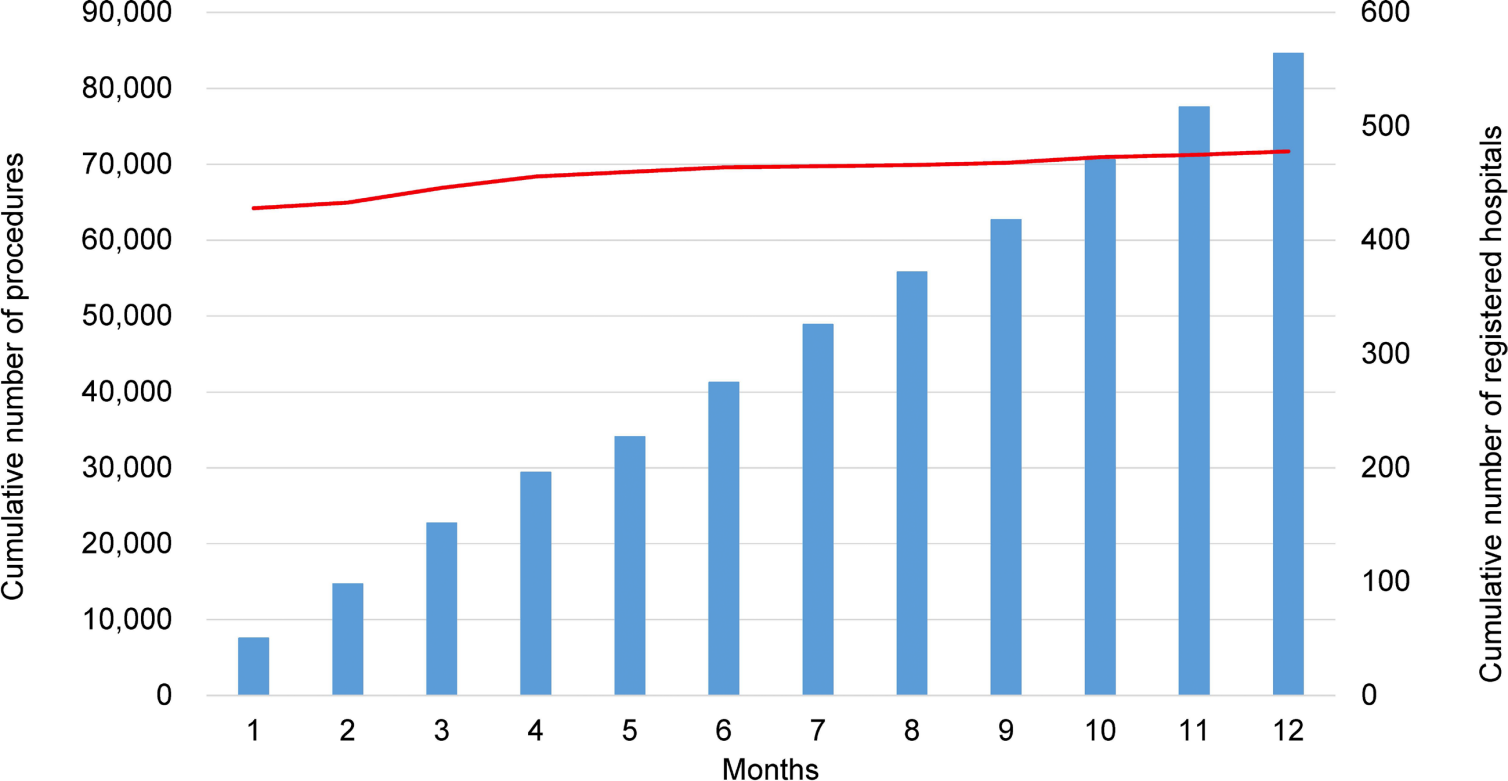
Cumulative number of registered hospitals (red line) and the patients (blue bars) during the year 2020.

**FIGURE 2:**
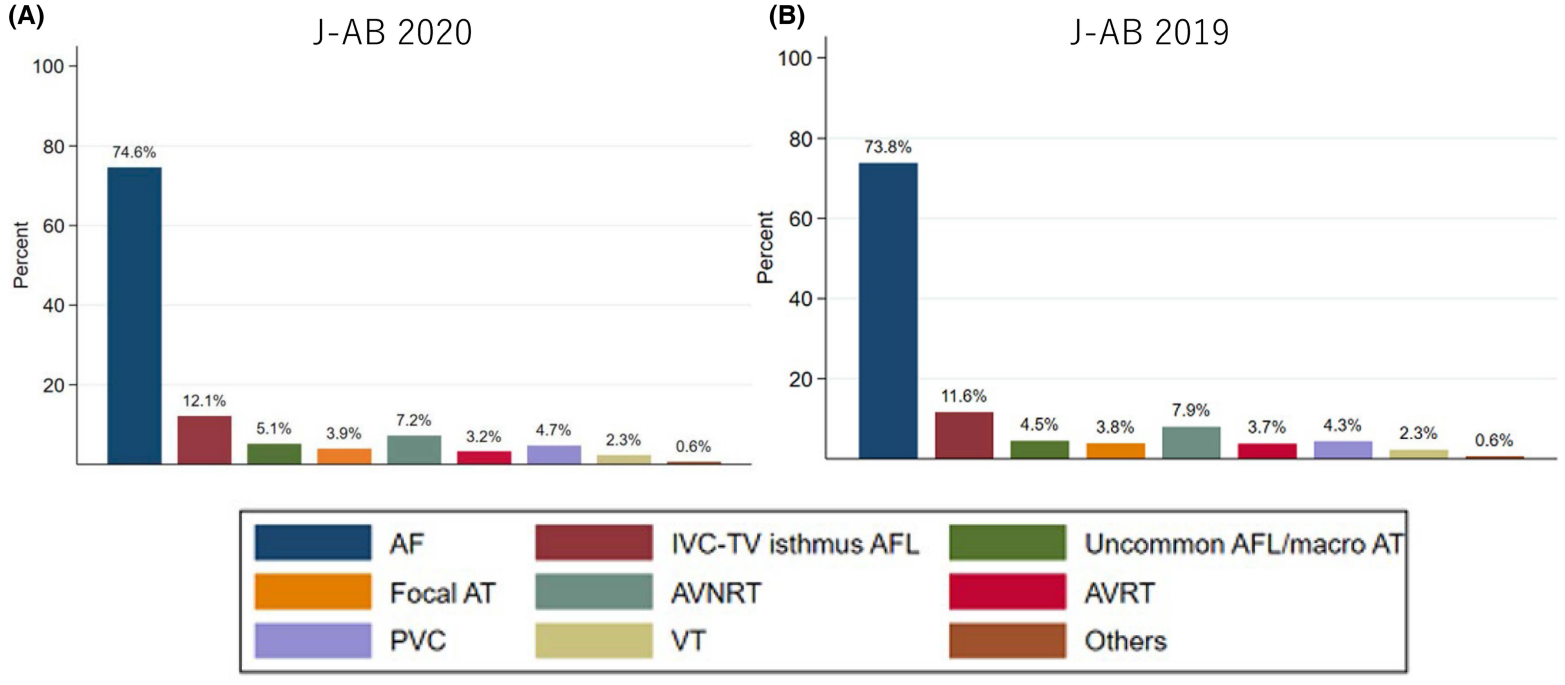
The number and rate of the target arrhythmias in the J‐AB registry 2020 (84, 591 procedures; A) and 2019 (80, 795 procedures; B). Abbreviations: AF, atrial fibrillation; AFL, atrial flutter; AT, atrial tachycardia; AVNRT, atrioventricular nodal reentrant tachycardia; AVRT, atrioventricular reentrant tachycardia; IVC, inferior vena cava; PVC, premature ventricular contraction; TV, tricuspid valve; VT, ventricular tachycardia.

**TABLE 1:** Patient characteristics

	All procedures	Atrial Fibrillation (AF)			Atrial flutter (AFL)/Atrial tachycardia (AT)			
		All AF	Paroxysmal AF (PAF)	Non‐PAF	All AFL/AT	IVC‐TV Isthmus dependent AFL	Uncommon AFL macro AT	Focal AT
*N*	84 591	63 096	36 352	26 573	14 985	9612	3746	2814
Age, mean ±SD	65.8 ± 13.1	67.4 ± 10.6	67.5 ± 10.9	67.2 ± 10.2	68.5 ± 12.5	68.6 ± 11.9	70.1 ± 11.5	66.2 ± 15.4
Gender, male	56 342 (66.6%)	43 696 (69.3%)	23 716 (65.2%)	19 871 (74.8%)	10 246 (68.4%)	7333 (76.3%)	2229 (59.5%)	1370 (48.7%)
Heart diseases	18 227 (21.6%)	13 020 (20.7%)	6593 (18.2%)	6415 (24.2%)	4747 (31.7%)	2920 (30.4%)	1700 (45.5%)	726 (25.8%)
IHD	6116 (7.2%)	4430 (7.0%)	2518 (6.9%)	1907 (7.2%)	1366 (9.1%)	976 (10.2%)	323 (8.6%)	168 (6.0%)
Cardiomyopathy	5080 (6.0%)	3591 (5.7%)	1422 (3.9%)	2168 (8.2%)	1148 (7.7%)	696 (7.2%)	389 (10.4%)	194 (6.9%)
Valve disease	3190 (3.8%)	1993 (3.2%)	947 (2.6%)	1040 (3.9%)	1429 (9.5%)	764 (7.9%)	711 (19.0%)	201 (7.1%)
CHD	1026 (1.2%)	502 (0.8%)	294 (0.8%)	208 (0.8%)	508 (3.4%)	300 (3.1%)	228 (6.1%)	92 (3.3%)

	Ventricular tachycardia (VT)						
	Atrioventricular nodal reentrant tachycardia	Atrioventricular reentrant tachycardia	Premature ventricular contraction	Idiopathic VT	VT due to ischemic cardiomyopathy	VT due to nonischemic cardiomyopathy	VT due to CHD
*N*	6071	2723	3949	806	486	544	18
Age, mean ±SD	59.0 ± 16.8	48.2 ± 20.2	58.3 ± 16.1	54.6 ± 19.0	68.8 ± 10.5	64.3 ± 12.9	42.4 ± 18.8
Gender, male	2661 (43.8%)	1760 (64.6%)	2190 (55.5%)	517 (64.1%)	427 (87.9%)	434 (79.8%)	12 (66.7%)
Heart diseases	515 (8.5%)	193 (7.1%)	797 (20.2%)	156 (19.4%)	450 (92.6%)	494 (90.8%)	18 (100.0%)
IHD	157 (2.6%)	64 (2.4%)	301 (7.6%)	51 (6.3%)		28 (5.1%)	0 (0%)
Cardiomyopathy	63 (1.0%)	30 (1.1%)	310 (7.9%)	71 (8.8%)	12 (2.5%)		0 (0%)
Valve disease	74 (1.2%)	14 (0.5%)	83 (2.1%)	18 (2.2%)	22 (4.5%)	40 (7.4%)	0 (0%)
CHD	46 (0.8%)	33 (1.2%)	20 (0.5%)	11 (1.4%)	3 (0.6%)	2 (0.4%)	

Abbreviations: CHD, congenital heart disease; IHD, ischemic heart disease; SD, Standard Deviation.

**TABLE 2:** Acute outcomes

2020		2019		2020–2019
Pulmonary vein isolation of atrial fibrillation (*n* = 61 757)		Pulmonary vein isolation for atrial fibrillation (*n* = 58 429)		
Ablation system		Ablation system	*n* (%)	% change
RF alone	47 022 (76.14%)	RF alone	43 047 (73.67%)	+2.47%
Ballon alone (Cryo, hot, laser)	9953 (16.12%)	Balloon alone (Cryo, hot, laser)	10 464 (17.91%)	−1.79%
RF + Ballon combination	4419 (7.16%)	RF + Balloon combination	4586 (7.85%)	−0.69%
Others	172 (0.28%)	Others	168 (0.29%)	−0.01%
Missing	191 (0.31%)	Missing	164 (0.28%)	+0.03%
Patient with a first session	50 193	Patient with a first session	47 726	
Success	49 881 (99.38%)	Success	47 462 (99.45%)	−0.07%
Unsuccess	240 (0.48%)	Unsuccess	186 (0.39%)	+0.09%
Unknown	72 (0.14%)	Unknown*	18 (0.04%)*	+0.10%*
		Already isolated*	60 (0.13%)*	*
Patient with second session	9511	Patient with second session	8863	
Success	7688 (80.83%)	Success	7448 (84.03%)	−3.20%
Unsuccess	20 (0.21%)	Unsuccess	19 (0.21%)	+0.00%
Already isolated	1756 (18.46%)	Already isolated	1388 (15.66%)	+2.80%
Unknown	47 (0.49%)	Unknown	8 (0.09%)	+0.40%
Additional ablation only	618 (6.09%)	Additional ablation only	577 (6.09%)	+0.00%
Patient with third session	2053	Patient with third session	2090	
Success	1191 (58.01%)	Success	1138 (64.40%)	−6.39%
Unsuccess	6 (0.29%)	Unsuccess	4 (0.23%)	+0.06%
Already isolated	850 (41.40%)	Already isolated	625 (35.37%)	+6.03%
Additional ablation only*	324 (13.61%)*	Additional ablation only	319 (15.26%)	−1.65%*
Unknown*	6 (0.29%)*			*
IV‐TV isthmus dependent atrial flutter (*n* = 9612)		IV‐TV isthmus dependent atrial flutter (*n* = 8838)		
Success	9544 (99.29%)	Success	8776 (99.30%)	−0.01%
Unsuccess	66 (0.69%)	Unsuccess	59 (0.67%)	+0.02%
Unknown	2 (0.02%)	Unknown	3 (0.03%)	−0.01%
Uncommon atrial flutter/ atrial tachycardia (*n* = 3746)		Uncommon atrial flutter/atrial tachycardia (*n* = 3132)		
Complete success	3198 (85.37%)	Complete success	2650 (84.61%)	+0.76%
Partial success	356 (9.50%)	Partial success	319 (10.19%)	−0.69%
Unsuccess	145 (3.87%)	Unsuccess	103 (3.29%)	+0.58%
Unknown	47 (1.25%)	Unknown	60 (1.92%)	−0.67%
Focal atrial tachycardia (*n* = 2814)		Focal atrial tachycardia (*n* = 2686)		
Complete success	2354 (83.65%)	Complete success	2238 (83.32%)	+0.33%
Partial success	311 (11.05%)	Partial success	313 (11.65%)	−0.60%
Unsuccess	107 (3.80%)	Unsuccess	101 (3.76%)	+0.04%
Unknown	42 (1.49%)	Unknown	34 (1.27%)	+0.22%
Atrioventricular nodal reentrant tachycardia by slow‐fast (n=5,247)*		Atrioventricular nodal reentrant tachycardia by slow‐fast (*n* = 5574)		
Complete success	5127 (97.71%)	Complete success	5457 (97.90%)	−0.19%
Partial success	74 (1.41%)	Partial success	70 (1.26%)	+0.15%
Unsuccess	32 (0.61%)	Unsuccess	29 (0.52%)	+0.09%
Unknown	14 (0.27%)	Unknown	18 (0.32%)	−0.05%
Atrioventricular nodal reentrant tachycardia by fast‐slow (*n* = 531)		Atrioventricular nodal reentrant tachycardia by fast‐slow (*n* = 581)		
Complete success	502 (94.54%)	Complete success	558 (96.04%)	−1.50%
Partial success	24 (4.52%)	Partial success	18 (3.10%)	+1.42%
Unsuccess	2 (0.38%)	Unsuccess	3 (0.52%)	−0.14%
Unknown	3 (0.56%)	Unknown	2 (0.34%)	+0.22%
Atrioventricular nodal reentrant tachycardia by slow‐slow (*n* = 326)				
Complete success	314 (96.32%)			
Partial success	7 (2.15%)			
Unsuccess	3 (0.92%)			
Unknown	2 (0.61%)			
Atrioventricular nodal reentrant tachycardia by other (*n* = 103)		Atrioventricular nodal reentrant tachycardia by other (*n* = 581)		
Complete success	86 (83.50%)	Complete success	339 (90.40%)	
Partial success	10 (9.71%)	Partial success	20 (5.33%)	
Unsuccess	3 (2.91%)	Unsuccess	7 (1.87%)	
Unknown	4 (3.88%)	Unknown	9 (2.40%)	
Atrioventricular reentrant tachycardia by kent (*n* = 2672)		Atrioventricular reentrant tachycardia by kent (*n* = 2951)		
Complete success	2589 (96.89%)	Complete success	2840 (96.24%)	+0.65%
Unsuccess	68 (2.54%)	Unsuccess	85 (2.88%)	−0.34%
Unknown	15 (0.56%)	Unknown	26 (0.88%)	−0.32%
Premature ventricular contraction (*n* = 3949)		Premature ventricular contraction (*n* = 3501)		
Complete success	3031 (76.75%)	Complete success	2642 (75.46%)	+1.29%
Partial success	658 (16.66%)	Partial success	602 (17.20%)	−0.54%
Unsuccess	216 (5.47%)	Unsuccess	228 (6.51%)	−1.04%
Unknown	44 (1.11%)	Unknown	29 (0.83%)	+0.28%
Idiopathic ventricular tachycardia (*n* = 806)		Idiopathic ventricular tachycardia (*n* = 781)		
Complete success	628 (77.92%)	Complete success	595 (76.18%)	+1.74%
Partial success	134 (16.63%)	Partial success	122 (15.62%)	+1.01%
Unsuccess	28 (3.47%)	Unsuccess	42 (5.38%)	−1.91%
Unknown	16 (1.98%)	Unknown	22 (2.82%)	−0.84%
Ventricular tachycardia due to ischemic cardiomyopathy (*n* = 486)		Ventricular tachycardia due to ischemic cardiomyopathy (*n* = 433)		
Complete success	342 (70.37%)	Complete success	272 (62.82%)	+7.55%
Partial success	111 (22.84%)	Partial success	117 (27.02%)	−4.18%
Unsuccess	21 (4.32%)	Unsuccess	20 (4.62%)	−0.30%
Unknown	12 (2.47%)	Unknown	24 (5.54%)	−3.07%
Ventricular tachycardia due to nonischemic cardiomyopathy (*n* = 544)		Ventricular tachycardia due to nonischemic cardiomyopathy (*n* = 502)		
Complete success	295 (54.23%)	Complete success	289 (57.57%)	−3.34%
Partial success	177 (32.54%)	Partial success	156 (31.08%)	+1.46%
Unsuccess	48 (8.82%)	Unsuccess	40 (7.97%)	+0.85%
Unknown	24 (4.41%)	Unknown	17 (3.39%)	+1.02%
Ventricular tachycardia due to CHD (n=18)*		Ventricular tachycardia due to CHD (*n* = 18)		
Complete success	15 (83.33%)	Complete success	10 (55.56%)	+27.77%
Partial success	2 (11.11%)	Partial success	7 (38.89%)	−27.78%
Unsuccess	1 (5.56%)	Unsuccess	1 (5.56%)	+0.00%

* [Correction added on 22 September 2022 after first online publication: The values in the table 2 are amended.]

Abbreviations: CHD, congenital heart disease; IVC, inferior vena cava; RF, radiofrequency ablation; TV, tricuspid valve.

**TABLE 3:** Acute complications

Factor	2020		2019		2020–2019	
					%change	
	All patient	AF	All patient	AF	All patient	AF
*N*	84 591	63 096	80 795	59 624		
Complications during hospitalization	1992 (2.35%)	1578 (2.50%)	2023 (2.50%)	1633 (2.74%)	−0.15%	−0.24%
Major bleeding (BARC ≧ 2)	776 (0.92%)	567 (0.90%)	902 (1.12%)	700 (1.17%)	−0.20%	−0.27%
Cardiac tamponade	490 (0.58%)	335 (0.53%)	532 (0.66%)	380 (0.64%)	−0.08%	−0.11%
Embolism	141 (0.17%)	126 (0.20%)	149 (0.18%)	128 (0.21%)	−0.01%	−0.01%
Phrenic nerve paralysis	254 (0.30%)	245 (0.39%)	212 (0.26%)	205 (0.34%)	+0.04%	+0.05%
Esophagus	99 (0.12%)	98 (0.16%)	147 (0.18%)	146 (0.24%)	−0.06%	−0.08%
Esophagus ulcer	19 (0.02%)	19 (0.03%)	20 (0.02%)	19 (0.03%)	+0.00%	+0.00%
Gastric hypomotility	82 (0.10%)	81 (0.13%)	127 (0.16%)	127 (0.21%)	−0.06%	−0.08%
Atrioesophageal fistula	0 (0)	0 (0)	0 (0)	0 (0)	−0.00%	−0.00%
Pericarditis	110 (0.13%)	91 (0.14%)	99 (0.12%)	84 (0.14%)	+0.01%	+0.00%
Sick sinus syndrome	152 (0.18%)	117 (0.19%)	134 (0.17%)	110 (0.18%)	+0.01%	+0.01%
Atrioventricular block	68 (0.08%)	26 (0.04%)	65 (0.08%)	17 (0.03%)	+0.00%	+0.01%
Death during hospitalization	92 (0.11%)	33 (0.05%)	89 (0.11%)	34 (0.06%)	+0.00%	−0.01%
Cardiac death	54 (0.06%)	14 (0.02%)	58 (0.07%)	18 (0.03%)	−0.01%	−0.01%
Related to ablation therapy	3 (0.004%)	0 (0)	2 (0.002%)	1 (0.002%)	+0.00%	+0.00%
Non cardiac death	38 (0.04%)	19 (0.03%)	31 (0.04%)	16 (0.03%)	+0.00%	+0.00%
Related to ablation therapy	2 (0.002%)	2 (0.003%)	1 (0.001%)	0 (0)	+0.00%	+0.00%

## FUNDING INFORMATION

This work was supported by Japanese Heart Rhythm Society.

## CONFLICT OF INTEREST

Kengo Kusano: Speaker honoraria from DAIICHI SANKYO COMPANY, Ltd., Nippon Boehringer Ingelheim, Biotronik Japan, Bayer Yakuhin, Pfizer, and Medtronic Japan, and research grants from Medtronic Japan, HITACHI, Biotronic Japan, Mebix, and JSR. Teiichi Yamane: Speaker honoraria from DAIICHI SANKYO COMPANY, Ltd., Medtronic Japan, and BEG Company, Ltd, and research grants from Nippon Boehringer Ingelheim. Koichi Inoue: Speaker honoraria from DAIICHI SANKYO COMPANY, Ltd., Bristol Myers Squibb, Bayer Yakuhin, Nippon Boehringer Ingelheim, Johnson & Johnson KK, Medtronic Japan, and Boston Scientific Japan. Koji Miyamoto received research fundings irrelevant to this study from Abbott, Japan Lifeline, and lecture fees from Abbott, Nihon‐koden, Johnson & Johnson KK, Medtronic Japan, Japan Lifeline, Nippon Boehringer Ingelheim, DAIICHI SANKYO COMPANY, Ltd., Brystol Myer Squibb, Pfizer, Bayer Yakuhin. Seiji Takatsuki received research fundings irrelevant to this study from Nippon Boehringer Ingelheim, Japan Lifeline, Eizai, Boston Scientific Japan, Johnson & Johnson KK and lecture fees from Medtronic Japan, Japan Lifeline, DAIICHI SANKYO COMPANY, Ltd., Pfizer, Boston Scientific Japan, Bayer Yakuhin, Biotronik Japan, Nippon Boehringer Ingelheim, Brystol Myers Squibb, Nihon‐koden. Wataru Shimizu: Research grant from DAIICHI SANKYO COMPANY, Ltd., and Nippon Boehringer Ingelheim, and Speaker honoraria from DAIICHI SANKYO COMPANY, Ltd., Bristol Myers Squibb, Bayer Yakuhin, Nippon Boehringer Ingelheim, Ono Pharmaceutical Co, Ltd, Otsuka Pharmaceutical Co, Ltd, Novartis Pharma K.K., and Medtronic Japan. None: M.T., Y.M.N, M.K, M.N, K.K, R.T, Y.I, K.N.

## ETHICAL STATEMENT

This study was approved by the Institutional Review Board (IRB) of the National Cerebral and Cardiovascular Center (M28‐114‐7, approved on Dec 21, 2016), Japan, along with the IRBs of all participating hospitals.
